# Factors associated with bronchiectasis in patients with uncontrolled asthma; the NOPES score: a study in 398 patients

**DOI:** 10.1186/s12931-018-0746-7

**Published:** 2018-03-16

**Authors:** A. Padilla-Galo, C. Olveira, L. Fernández de Rota-Garcia, I. Marco-Galve, A. J. Plata, A. Alvarez, F. Rivas-Ruiz, A. Carmona-Olveira, J. J. Cebrian-Gallardo, M. A. Martinez-Garcia

**Affiliations:** 1 Pneumology Unit. Agencia Sanitaria Costa del Sol, Carretera Nacional 340, Km 187, 29603 Marbella, Málaga Spain; 2 Pneumology Department, IBIMA (Institute for biomedical research of Málaga), Hospital Regional Universitario de Málaga/ University of Málaga, Avenida Carlos Haya, 29010 Málaga, Spain; 3 Radiology Deparment. Hospital de Alta Resolución de Benalmádena, Arroyo Hondo s/n, 29639 Benalmádena, Málaga Spain; 4 Infectious Diseases Department, Hospital Regional Universitario de Málaga, Avenida Carlos Haya, 29010 Málaga, Spain; 5 Research Unit. Red de Investigación en Servicios de Salud en Enfermedades Crónicas, REDISSEC (Spanish healthcare network for chronic diseases), Agencia Sanitaria Costa del Sol, Carretera Nacional 340, Km 187, 29603 Marbella, Málaga Spain; 6 Faculty of Medicine, University of Málaga, Málaga, Spain; 7 Pneumology Department, Hospital Universitario La Fe, Valencia, Spain

**Keywords:** Asthma, Bronchiectasis, Prevalence, Risk factors, NOPES

## Abstract

**Background:**

Some studies have reported a high prevalence of bronchiectasis in patients with uncontrolled asthma, but the factors associated with this condition are unknown. The objective of this study was to determine the prevalence of bronchiectasis in uncontrolled moderate-to-severe asthma and to identify risk factors and their correlation with bronchiectasis in these patients.

**Methods:**

This is a prospective study of data from consecutive patients with uncontrolled moderate-to-severe asthma. Diagnosis of bronchiectasis was based on high-resolution computed tomography. A prognostic score was developed using a logistic regression model, which was used to determine the factors associated with bronchiectasis.

**Results:**

A total of 398 patients (60% with severe asthma) were included. The prevalence of bronchiectasis was 28.4%. The presence of bronchiectasis was associated with a higher frequency of chronic expectoration (OR, 2.95; 95% CI, 1.49–5.84; *p* = 0.002), greater severity of asthma (OR, 2.43; 95% CI, 1.29–4.57; *p* = 0.006), at least one previous episode of pneumonia (OR, 2.42; 95% CI, 1.03–5.69; *p* = 0.044), and lower levels of FeNO (OR, 0.98; 95% CI, 0.97–0.99; *p* = 0.016). The NOPES score was developed on the basis of these variables (Fe**NO**[cut off point 20.5 ppb], **P**neumonia, **E**xpectoration and asthma **S**everity), and it ranges from 0 to 4 points, where 0 means “no risk” and 4 corresponds to “high risk”. The NOPES score yielded an AUC-ROC of 70% for the diagnosis of bronchiectasis, with a specificity of 95%.

**Conclusions:**

Almost a third of the patients with uncontrolled moderate-to-severe asthma had bronchiectasis. Bronchiectasis was related to the severity of asthma, the presence of chronic expectoration, a previous history of pneumonia, and lower levels of FeNO. The NOPES score is an easy-to-use scoring system with a high prognostic value for bronchiectasis in patients with uncontrolled moderate-to-severe asthma.

## Background

Asthma is a heterogeneous condition characterized by chronic inflammation of the pulmonary airways [1] that currently afflicts 300 million people worldwide [2].

Bronchiectasis (BE) is defined as bronchial dysfunction secondary to an infectious, inflammatory or reparative process in the airways that permanently damages the bronchial walls and causes irreversible enlargement of the airways. Although the real prevalence of BE is unknown, it has been estimated as being anywhere between 42 and 566 cases per 100,000 people and it particularly affects women and the elderly, but a significant trend toward under-diagnosis has also been acknowledged [3, 4]. However, the number of diagnoses is quickly rising, due, among other factors, to the population’s greater longevity, the greater chronicity of the diseases that trigger BE and, above all, the greater reliability of the high-definition tomographic techniques now in use [5]. Thus, its incidence and prevalence are increasing, particularly in older age groups, and it is associated with a marked increase in mortality [6], making it probably the third most frequent chronic inflammatory airway disorder [5].

Asthma and bronchiectasis are different conditions that frequently coexist. Furthermore, in our experience, a diagnosis of bronchiectasis in asthma patients could lead to modifications to both therapy and prognosis (as in the case of COPD patients [7–9]). Most studies of the prevalence or characteristics of bronchiectasis in asthmatic patients are retrospective [10–14], involving a small sample [15–18], and including biases such as smoking [10, 18, 19], or allergic bronchopulmonary aspergillosis (ABPA) [10–12, 17], while high-resolution CT scanning (HRCT) was not always performed [11, 13, 14]. This study sought to determine the prevalence of bronchiectasis in non-smokers with uncontrolled moderate-to-severe asthma (UMSA), based on the largest sample ever used and on HRCT findings. Another objective was to identify factors associated with the presence of bronchiectasis in these patients.

## Methods

### Study population

This prospective study included consecutive patients (*n* = 432) with UMSA (according to GINA criteria [Global Initiative for Asthma] steps 3, 4 and 5 [1]) during a period of 3 years at the Asthma Unit of the Hospital Costa del Sol, Spain. All patients were diagnosed by objective tests (FEV_1_ reversibility ≥12%, positive results on methacholine or FEV_1_ variability ≥20%). We classified patients as moderate or severe: moderate if they need at least 100 μg fluticasone propionate equivalent per day and additional controller drugs, or at least 250 μg fluticasone propionate equivalent per day without additional controller drugs; and severe if they need at least 1000 μg fluticasone propionate equivalent per day and required additional controller drugs.

**Fig. 1:**
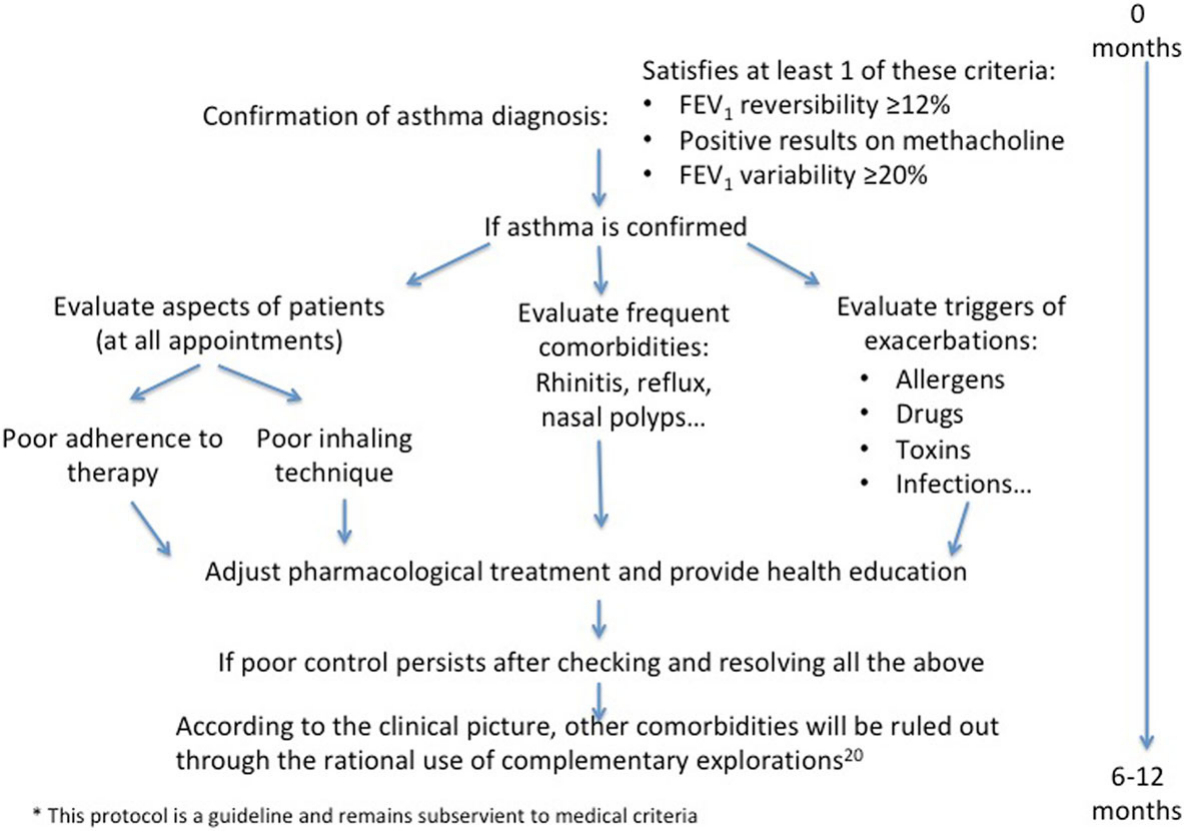
Protocol for the management of uncontrolled asthma in the Hospital Costa del Sol

We used a standardized protocol to try to improve the control of these patients. This was designed to ensure patients’ adherence to both the therapy and the appropriate inhaler procedures, provide health education, adjust the treatment and rule out other comorbidities. If, after all this, and after at least a year of follow-up (as required for inclusion in the study), the disease was still not satisfactorily under control, then a high-resolution computed tomography chest scan (HRCT) was carried out to evaluate other pathologies, in accordance with the main guidelines for asthma [1, 20]. See Fig. 1. Uncontrolled was defined (during the clinical interview held in the consulting room after at least 1 year of follow-up) as, at least one of the following: daytime asthma symptoms > 2/week, reliever needed for symptoms > 2/week, waking up in the night due to asthma or any activity limitation due to asthma [1].

At the following appointment, one to three months after inclusion in the study, those patients diagnosed as having BE by means of HRCT underwent a complete examination, following specific guidelines [3], to investigate the etiology of bronchiectasis, including alpha 1-antitrypsin deficiency, ABPA, cystic fibrosis, and immunodeficiency and systemic diseases. Patients with these conditions were excluded, because in this research we are looking for the presence of bronchiectasis related to asthma and no other associated pathologies. These patients were subsequently reexamined in the consulting room 6 months after inclusion in the study (or earlier, if required by usual clinical practice) and the monthly sputum samples that had been collected were checked.

Previous episodes of pneumonia reported by patients or their medical records were recorded. Patients with a previous diagnosis of bronchiectasis, smokers, and former smokers of more than 10 packs per year were excluded (to avoid the possibility of concomitant COPD diagnosis). On inclusion, patients were required to have been in a stable phase for at least 8 weeks (no respiratory tract infection), and all the tests were performed in this phase. Written informed consent was obtained from all participants. The study was approved by our hospital’s Ethics Committee.

### Diagnosis of bronchiectasis

A chest HRCT scan was performed on all the patients, and bronchiectasis was diagnosed according to the criteria established by Naidich et al. [21]. The extension of bronchiectasis was determined according to the number of pulmonary segments and lobes affected (lingula was considered an independent lobe), and structural damage according to the Bhalla score [22] and modified Bhalla score [23]. Two radiologists experienced in the diagnosis of bronchiectasis separately read all the HRCT scans, blind to the other researchers. In the event of any discrepancy (as occurred in 46 cases), a third radiologist, similarly expert in the diagnosis of bronchiectasis, made the final decision. Bronchiectasis only visible in a single pulmonary segment was not considered [19].

### Clinical and analytical variables

Full clinical histories from diagnosis to inclusion in the study were compiled in a database. A standardized protocol was applied for the prospective collection of sociodemographic data (age, gender), clinical profile (degree of severity [1], evolution of asthma, atopy, expectoration [24]), comorbidities, exacerbations, use of antibiotics and corticoid therapy, and basic blood test. Dyspnea was evaluated by means of the modified Medical Research Council Scale for Dyspnea [25]), and we divided patients into groups 0–2 and 3–4, according to their degree of dyspnea. We chose this cut-off point because dyspnea ≥3 is one of the prognostic factors for mortality on the FACED prognostic scale [26]. We used the asthma control test (ACT) questionnaire to evaluate the degree of control of asthma in the 4 weeks prior to the clinical interview. The ACT [27] is a self-administered questionnaire that is easy for patients to complete; it includes four symptom-relief questions plus a patient’s self-assessment of the level of control [1] in the last 4 weeks, with scores ranging from 5 (worst control) to 25 (total control), and it has been validated in Spanish [28, 29]. A patient was considered as presenting rhinitis when he or she referred to anterior or posterior rhinorrhea, sneezing, nasal blockage or congestion, and/or pruritus/itching in the nose. These symptoms had to be manifest for two or more consecutive days, for more than an hour on most days [30]. All patients were classified as having gastroesophageal reflux disease when he or she presented both a clinical picture suggestive of this disorder and a gastroscopy with esophageal injuries compatible with reflux, or via a pH metre showing evidence of a pathological reflux. Nasal polyposis was diagnosed by an otarhinolaryngologist via direct visualization of the polyps with rhinofibrolaryngoscopy. Patients were considered atopic when they had positive allergic prick tests or specific IgE positive to pneumo-allergens, whenever these positive findings also had clinical relevance. A patient was considered to have chronic expectoration when he or she satisfied the clinical criteria for chronic bronchitis (coughing and expectoration for 3 months in at least the last 2 years, without attribution to any other cause or disease). The purulence of the sputum was evaluated by the Murray scale [24] on the basis of direct observation of the sample. The comorbidities were evaluated by means of the Charlson index [31], which assesses life expectancy 10 years henceforth, depending on the patient’s age and comorbidities at the time of the evaluation. We also used two prognostic scales for BE: FACED [26] and the BSI index score [32].

### Fractional exhaled nitric oxide and spirometry values

Fractional exhaled nitric oxide (FeNO) was measured with a conventional chemoluminescence analyser (NIOX, Aerocrine AB, Sweden) using the online standardized single-breath technique, following international guidelines [33]. Spirometry (Jaeger Oxycon Pro® Erich Jaeger, Germany) was then performed, following international guidelines [34].

### Exacerbations

Moderate-to-severe exacerbations were recorded according to standard guidelines [35]. All the patients were trained to identify exacerbation symptoms and instructed to visit their family doctor or outpatient or hospital ED following any deterioration; they were also asked to record details about their condition and prescriptions (antibiotics and systemic steroids). This information was confirmed in their medical records.

### Sputum culture

All patients underwent a monthly microbiological analysis of spontaneous morning sputum during the first 6 months. Instructions were given to ensure that sputum was collected correctly, with low percentages of saliva recorded [36]. Diluted secretions were plated on chocolate, blood, and McConkey and Sabouraud agar. A cut-off point of ≥10^3^ was set for the identification of abnormal cultures positive for PPM, following published methods [37–39]. The presence of a single potentially pathogenic microorganism (PPM) in three different monthly sputum samples, without any concurrent antibiotic treatment, was considered chronic colonization [40].

### Statistical analysis

All data were analyzed using R commander [41] and expressed as means and standard deviation for quantitative variables, and as absolute values and percentages for qualitative variables.

The kappa statistic (*k* value) was calculated for assessment of inter-reader agreement for qualitative radiological variables (presence of bronchiectasis and presence of bronchial wall thickening).

Bivariate analysis based on Student *t*-test and Chi-squared test was performed, using bronchiectasis as outcome variable. Multivariate logistic regression was performed with the same outcome variable. The variables considered by the authors to be of clinical interest (gender and use of oral corticoids), as well as those variables that presented statistically significant differences in bivariate analysis, were included as independent variables in the first step. The forward technique (Wald test) was used to remove variables with a *p* > 0.1 from the logistic model (conditioned by selecting the model with the fewest variables and strongest goodness of fit), as well as to evaluate any possible interactions between independent variables. ORs and 95% CIs were calculated for independent variables. Together with the above-mentioned risk indicators, the goodness of fit was assessed using the Hosmer-Lemeshow test, and the variance of the model was explained by Nagelkerke R^2^.

Receiver operating characteristic (ROC) curves were obtained to elucidate the clinical functions of FeNO in the diagnosis of bronchiectasis in asthmatic patients. An AUC-ROC≥0.70 is established as a suitable diagnostic performance [42], and the optimal cut-off point was established by means of the Youden Index.

Finally, in order to evaluate the effectiveness of the NOPES score as regards the presence of bronchiectasis for the cut-off points of ≥1, ≥2 and ≥ 3, the basic indicators [43, 44] of the diagnostic tests were calculated in 2 × 2 Tables. *P* < 0.05 was considered significant.

## Results

### Clinical characteristics and prevalence

**Fig. 2:**
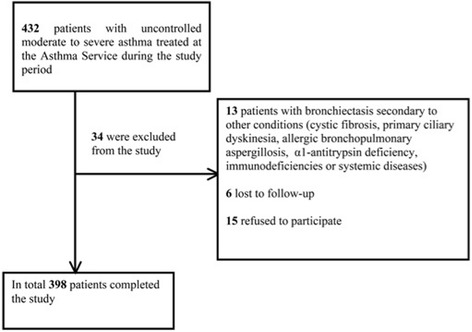
Flow diagram

A total of 432 patients with UMSA were recruited consecutively and prospectively during the study period. Fifteen patients refused to participate, 13 were excluded because they presented bronchiectasis secondary to another disease, and six were lost to follow-up (Fig. 2). Of the remaining 398 patients, 160 (40.2%) had moderate asthma and 238 (59.8%) had severe asthma. In total, 20.6% of patients with moderate asthma had bronchiectasis but its prevalence was substantially higher in those with severe asthma (33.6%, *p* < 0.001).

The inter-reader kappa values for the diagnosis of bronchiectasis and bronchial wall thickening were k = 0.85 and k = 0.73, respectively.

Table 1 shows the basic characteristics of the identified cases of bronchiectasis. Tables 2 and 3 show clinical, functional, and analytical data for asthma patients with and without bronchiectasis. Patients with bronchiectasis were older, had more severe asthma and more chronic expectoration, purulent sputum, and exacerbations, and they used more health resources. In total, 133 patients (33.4%) presented at least one valid culture. No significant differences were found between the two groups regarding the presence of PPM.

**Table 1:** Bronchiectasis characteristics and other CT scan findings in patients with asthma and bronchiectasis

Characteristics	
Patients with bronchiectasis (*n* = 113)	28.4%
Location	
Only upper lobes (*n* = 6)	5.3%
Only lingula or middle lobule (*n* = 8)	7.1%
Only lower lobes (*n* = 28)	24.8%
Only right (*n* = 20)	17.7%
Only left (*n* = 10)	8.8%
Bilateral (*n* = 83)	73.5%
Central bronchiectasis (*n* = 1)	0.9%
Extension	
Localized (only one lobule) (*n* = 21)	18.6%
Disseminated (four or more lobules) (*n* = 39)	34.5%
No. affected lobules (mean ± SD)	2.97 ± 1.5
Type^a^	
Cylindrical (*n* = 105)	92.9%
Cystic (n = 8)	7.1%
Thickening of bronchial wall^b^	
Slight (*n* = 96)	84.96%
Moderate (*n* = 17)	14.04%
Severe (*n* = 0)	0%
Other findings	
Expiratory flow limitation (*n* = 25)	22.1%
Atelectasis (*n* = 70)	61.9%
Radiological signs of pulmonary hypertension (*n* = 2)	1.8%
Mucous plugs (*n* = 89)	78.8%
Bronchiolitis signs (*n* = 69)	61.1%
Chronic interstitial fibrosis (*n* = 1)	0.9%
Adenopathies (> 10 mm) (*n* = 0)	0%
Radiological scales	
Bhalla (mean ± SD)	16.97 ± 2.76
Modified Bhalla (mean ± SD)	16.74 ± 2.8
Prognostic scales	
FACED (mean ± SD)	1.45 ± 1.21
BSI (mean ± SD)	4.82 ± 2.98

Data are presented as n (number) and (%) for qualitative variables and mean (standard deviation) for quantitative variables

^a^ Some patients had both cylindrical and cystic bronchiectasis

^b^ Slight thickening is less than the diameter of the adjacent vessel; moderate, similar to the diameter of the adjacent vessel; severe, greater than the diameter of the adjacent vessel

**Table 2:** Baseline and clinical characteristics of subjects with asthma, with and without bronchiectasis

Parameter	Whole group (*n* = 398)	Asthma with bronchiectasis (*n* = 113)	Asthma without bronchiectasis (*n* = 285)	*P* value
Age, years (m ± sd)	57.06 ± 15.6	59.83 ± 13.8	55.96 ± 16.1	0.017
Female gender, n (%)	281 (70.6)	87 (77)	194 (68.1)	ns
BMI (m ± sd)	28.77 ± 5.3	29.04 ± 5.4	28.7 ± 5.2	ns
Dyspnea MRC,				ns
Score 0–2, n (%)	370 (93.7)	102 (91.9)	268 (94.4)	
Score 3–4, n (%)	25 (6.3)	9 (8.1)	16 (5.6)	
Packs/yr smoked (m ± sd)	1.54 ± 2.85	1.29 ± 2.62	1.64 ± 2.93	ns
Never smoked, n (%)	283 (71.1)	85 (75.2)	198 (69.5)	ns
Asthma by severity:				0.005
Moderate Asthma, n (%)	160 (40.2)	33 (29.2)	127 (44.6)	
Severe Asthma, n (%)	238 (59.8)	80 (70.8)	158 (55.4)	
ACT (m ± sd)	15.36 ± 4.4	14.6 ± 4.7	15.7 ± 4.3	0.06
Time from diagnosis of asthma in years (m ± sd)	16.8 ± 16	18.1 ± 16.6	16.3 ± 15.8	ns
Previous pneumonia, n (%)	63 (15.8)	26 (23)	37 (13)	0.020
Rhinitis, n (%)	239 (60.1)	62 (56.9)	177 (63.4)	ns
Nasal sinus polyps, n (%)	46 (11.7)	19 (17.1)	27 (27.8)	0.059
GERD, n (%)	112 (28.3)	33 (29.5)	79 (27.8)	ns
Atopy, n (%)	203 (54.6)	52 (48.6)	151 (57)	ns
Corticoid-dependent, n (%)	25 (6.3)	9 (8)	16 (5.6)	ns
ED visits in the last year (m ± sd)	1.85 ± 2.99	2.34 ± 3.4	1.66 ± 2.8	0.042
Hospitalizations in the last year (m ± sd)	0.13 ± 0.39	0.13 ± 0.39	0.12 ± 0.38	ns
Moderate-severe exacerbations (m ± sd)	2 ± 2.84	2.54 ± 3.35	1.79 ± 2.59	0.034
Cycles of oral corticoid therapy in the previous year (m ± sd)	1.1 ± 2.08	1.33 ± 2.33	1 ± 1.97	ns
Cycles of antibiotic therapy in the previous year (m ± sd)	1.35 ± 1.85	1.83 ± 2	1.16 ± 1.7	0.002
Chronic expectoration, n (%)	82 (20.6)	36 (31.9)	46 (16.1)	0.001
Purulent sputum, n (%)	16 (4)	9 (8)	7 (2.5)	0.020
Charlson Index (m ± sd)	3.18 ± 2.12	3.36 ± 1.8	3.1 ± 2.2	ns

Data are presented as n (number) and (%) for qualitative variables and mean (standard deviation) for quantitative variables

*MRC* Medical Research Council, *ns* not significant, *BMI* Body Mass Index, *ACT* Asthma control test, *GERD* Gastroesophageal reflux disease

**Table 3:** Functional, analytic, microbiologic, and radiological characteristics of subjects with asthma, with and without bronchiectasis

Parameter	Whole group (*n* = 398)	Asthma with bronchiectasis (*n* = 113)	Asthma without bronchiectasis (*n* = 285)	*P* value
FEV_1_, % Post-BD (m ± sd)	80 ± 22.9	77 ± 22	81 ± 23	0.061
FEV_1_, (ml) Post-BD (m ± sd)	1989 ± 829	1792 ± 785	2066 ± 834	0.002
FVC, % Post-BD (m ± sd)	93 ± 20	90 ± 17	94 ± 20	0.054
FVC, (ml) Post-BD (m ± sd)	2790 ± 1022	2542 ± 963	2888 ± 1029	0.002
FEV_1_/FVC ratio Post-BD (m ± sd)	69 ± 11	68.9 ± 11	69.8 ± 11	ns
Classification by FEV_1_%				ns
> 80%, n (%)	189 (47.59)	45 (39.8)	144 (50.5)	
50- < 80%, n (%)	179 (45)	56 (49.6)	123 (43.2)	
< 50%, n (%)	30 (7.5)	12 (10.6)	18 (6.3)	
FeNO, ppb (m ± sd)	29.9 ± 29.9	23.9 ± 23	32.3 ± 32	0.039
Eosinophils, cells/μL (m ± sd)	310 ± 254	324 ± 288	304 ± 239	ns
IgE, IU/mL (m ± sd)	269 ± 541	205 ± 362	298 ± 609	ns
Positive sputum culture, n (%)	50 (24.2)	22 (29.7)	28 (21.1)	ns
Air trapping measured by HRCT, n (%)	52 (13)	25 (22.1)	27 (9.5)	0.002

Data are presented as n (number) and (%) for qualitative variables and mean (standard deviation) for quantitative variables; *ns* not significant, *FEV*_*1*_ Forced expiratory volume in one second, *post-BD* Post-bronchodilator, *FVC* Forced vital capacity, *FeNO* Fractional exhaled nitric oxide, *ppb* parts per billion

### Factors associated with the presence of bronchiectasis

The variables selected to enter the first phase in the logistic regression model were: age, gender, severity of asthma, chronic expectoration, purulence in the sputum, previous pneumonia, FeNO levels, presence of air trapping, use of oral corticoids and antibiotics in the year prior to inclusion in the study and presence of exacerbations in the year prior to inclusion in the study; among these variables, only severity of asthma, presence of chronic expectoration, previous pneumonia, and the FeNO value presented any independent association with the presence of bronchiectasis (Table 4).

**Table 4:** Logistic regression. Factors associated with the presence of bronchiectasis in patients with uncontrolled moderate-to-severe asthma

	ß	OR	95% CI	*p*
Severity of asthma	0.887	2.43	1.29–4.57	0.006
Chronic expectoration	1.082	2.95	1.49–5.84	0.002
Previous pneumonia	0.882	2.42	1.03–5.69	0.044
FeNO	−0.016	0.98	0.97–0.99	0.016

*FeNO* Fractional exhaled Nitric Oxide

Adjusted by: age, gender, purulence in the sputum, presence of air trapping, use of oral corticoids in the previous year, use of antibiotics in the previous year and presence of exacerbations in the previous year.

Nagelkerke’s R Square: 0.145.

Hosmer & Lemeshow test: *p* = 0.883.

### Prognostic score for the presence of bronchiectasis in patients with UMSA (NOPES score)

A score called NOPES (after Fe**NO**, **p**neumonia, **e**xpectoration and **s**everity) was developed, using logistic regression (Table 5). Of the 11 variables that were initially selected, only those with a statistically significant capacity to predict the presence of BE were chosen for the final score. In order to establish the score’s values, the ß of the independent qualitative dichotomic variables were selected from the multivariate logistic regression model, and these were given a value of “1”, as the three ß were all close to this number (round value), in order to obtain a score that is easy to construct and interpret in everyday clinical practice. As regards the FeNO, given that this is a quantitative variable, a ROC curve was developed to define its optimal cut-off value, which was 20.5 parts per billion (ppb), with an ROC AUC of 0.61 and a negative predictive value of 81%. Following the same criteria, a value of “1” was attributed, in this case to patients with FeNO < 20.5. The score ranges from 0 to 4 (where 0 means “less severity” and 4, “high severity”).

**Table 5:** Prognostic score for the presence of bronchiectasis in patients with uncontrolled moderate-to-severe asthma, cut-off points of the dichotomized variables, and scoring of each variable (NOPES score)

	Points
Severity of asthma	
Moderate	0
Severe	1
Chronic bronchial expectoration	
No	0
Yes	1
Previous pneumonia	
No	0
Yes	1
FeNO	
> 20.5 ppb	0
≤ 20.5 ppb	1

*FeNO* Fractional exhaled nitric oxide, *ppb* parts per billion

**Fig. 3:**
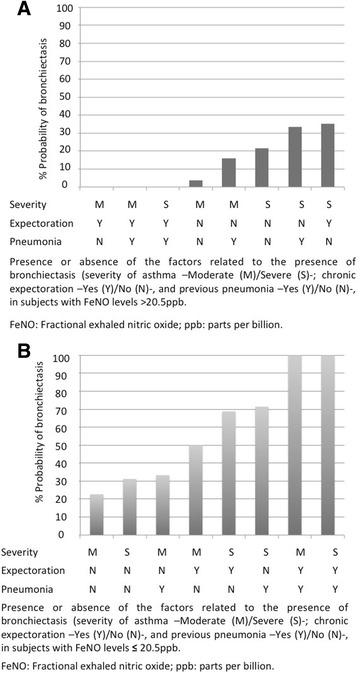
**a** Probability of bronchiectasis in patients with uncontrolled moderate-to-severe asthma and FeNO levels > 20.5 ppb. **b** Probability of bronchiectasis in patients with uncontrolled moderate-to-severe asthma with FeNO levels ≤20.5 ppb

Using the NOPES score, we calculated the probability of bronchiectasis, based on the presence or absence of different variables. The evaluation started with FeNO levels (Fig. 3a and b), meaning that a UMSA patient with FeNO levels > 20.5 ppb was unlikely to have bronchiectasis. Within the FeNO > 20.5 ppb group, the patients with severe asthma and chronic expectoration were most likely to have bronchiectasis (35%) (Fig. 3a). Subjects with FeNO ≤20.5 ppb were more likely to have bronchiectasis, however, and those with at least two further score variables—i.e. a score of 3— had a 69% probability of having bronchiectasis (Fig. 3b).

**Fig. 4:**
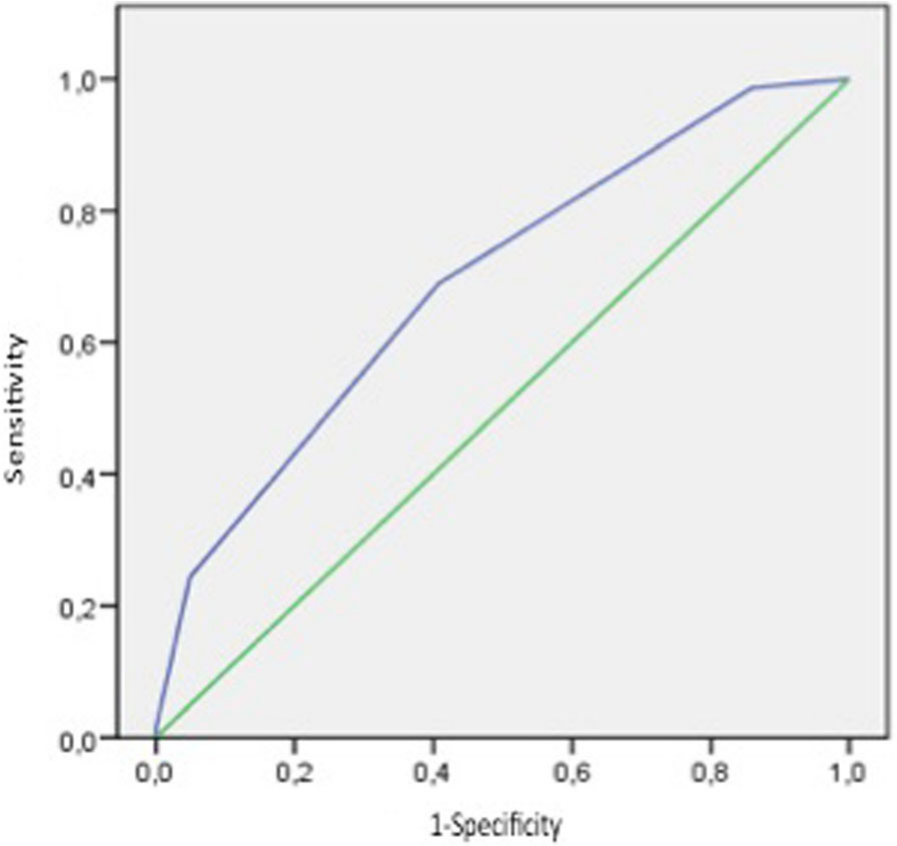
ROC curve for the NOPES score

**Fig. 5:**
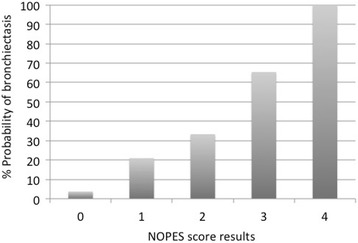
Probability of bronchiectasis in patients with uncontrolled moderate-to-severe asthma, according to the NOPES score

The AUC-ROC for the NOPES score was 0.7 (Fig. 4). Table 6 shows sensitivity, specificity, positive and negative predictive values, and prevalence of bronchiectasis according to the NOPES score (≥1, ≥2 and ≥ 3). According to the Youden Index, the best model was that based on NOPES scores ≥2. With a score of 3, this model showed excellent specificity (95%) and good negative (76%) and positive (67%) predictive values, obtaining 67% prevalence in UMSA patients. Figure 5 shows the probability of having bronchiectasis according to the NOPES score. If a UMSA patient has a score of 0, the likelihood of having bronchiectasis would be 3.7%, whereas in a patient with a score of 4 the likelihood would be 100%.

**Table 6:** NOPES score by cut-off point

NOPES score	≥1	≥2	≥3
Sensitivity (%)	98.7	68.9	24.3
Specificity (%)	14.1	59.2	95.1
Validity index (%)	38.4	62	74.8
Positive predictive value (%)	31.6	40.5	66.7
Negative predictive value (%)	96.3	82.6	75.8
Youden Index	0.13	0.28	0.19
Prevalence of BE (%)	31.6	40.8	66.7

*BE* Bronchiectasis

## Discussion

This study reveals a 28.4% prevalence of bronchiectasis in UMSA patients, 20.6% for moderate, and 33.6% for severe asthma.

According to the literature, the prevalence of bronchiectasis among patients with asthma ranges from 2.2% [14] to 77% [19]. Such discrepancies can be explained by inconsistencies in the methodologies used, as some studies included smokers [10, 18, 19], ABPA [10–12, 17], diseases related to bronchiectasis [45], and the inclusion of asthma patients with different degrees of severity [13, 15, 17, 18, 46], while not all patients underwent HRCT [11, 13, 14].

After demonstrating the effectiveness of HRCT in diagnosing bronchiectasis in ABPA, Neeld et al. [47] observed a high incidence of bronchiectasis in asthma patients and found cylindrical bronchiectasis in asthmatic patients without ABPA. Several authors have reported the presence of bronchiectasis in subjects with asthma and without ABPA [15, 46], supporting our findings. We also found other studies with similar percentages of BE in patients with asthma: Grenier et al. [18] and Khadadah et al. [16] conducted two studies in which they found a 28.5% prevalence of bronchiectasis in patients with asthma of varying degrees of severity. Gupta el al. [10] found a 40% prevalence of bronchiectasis in patients with severe asthma, although 5% met the criteria for ABPA, and the rate dropped to 26% when smokers were excluded. Menzies et al. [12] conducted a retrospective study of medical records that included patients meeting ABPA criteria and found a 35.3% prevalence of bronchiectasis.

In keeping with previous studies [15, 18, 46, 48], we found a higher prevalence of cylindrical (92.9%) and bilateral (73.5%) bronchiectasis, mainly in the lower lobes.

Multivariate analysis revealed that chronic bronchial expectoration was associated with bronchiectasis in UMSA. This variable has scarcely been investigated, although studies on bronchiectasis not associated with cystic fibrosis—such as that conducted by Goeminne et al. [49] — demonstrate that purulent sputum indicates the severity of inflammatory damage and the activity of proteolytic enzymes. Other studies of bronchiectasis not associated with cystic fibrosis have also found a relationship between the color of expectoration and the presence of PPM [24]. In our study, we observed that chronic bronchial expectoration and purulent sputum were more frequent in patients with asthma and bronchiectasis (31.9% vs 16.1%) compared to those with no bronchiectasis (8% vs. 2.5%). Chronic expectoration and purulent sputum are important factors for consideration in patients with asthma and bronchiectasis, especially given that they are not only correlated with *PPM* and a greater use of antibiotics but are also independent risk factors for the presence of bronchiectasis in asthmatic patients.

Another factor related to bronchiectasis was a history of pneumonia (a relationship known since the last century) [50].

Another predictor of bronchiectasis in UMSA is asthma severity. The results obtained are consistent with the literature [13, 15, 17, 18, 46] and show a higher prevalence of bronchiectasis in patients with severe asthma, compared to milder cases.

Our study’s contribution is the correlation found between higher FeNO levels and a lower probability of bronchiectasis. FeNO is a non-invasive biomarker of the inflammation of the airways in asthma; high levels are associated with eosinophilic inflammation of the airways [51–54]. Its concentration has been shown to be higher in patients with bronchial asthma than in a healthy population [55]. Moreover, numerous studies have demonstrated that FeNO values in asthma patients are associated with other characteristics of the disease, such as bronchial hyper-reactivity, the intensity of the symptoms, or the number of eosinophils in samples from the airways [51]. In this respect, FeNO has been defined as a biological marker of inflammation in asthma.

However, some studies of FeNO levels in BE have presented contradictory results. Thus, Kharitonov et al. [56] showed that high levels of FeNO in BE correlated with disease severity, as in the case of asthma, whereas Cho et al [57] found, in keeping with our results, that the FeNO levels in BE patients are lower than those found in asthma patients. Furthermore, BE is normally associated with neutrophilic inflammation [58, 59]. However, one recently published study [60] used induced sputum and FeNO in 40 BE patients as non-invasive measures of inflammation. These authors found that, compared with patients with BE and neutrophilic inflammation or paucigranulocytic phenotype, patients with BE and eosinophilic or mixed (neutrophilic-eosinophilic) inflammation had higher levels of FeNO and greater reversibility to bronchodilation, as in the case of asthma [51]. Their findings in other inflammatory parameters (IL-13 increased slightly in bronchiectasis, even in patients with eosinophilic inflammation) led them to establish the hypothesis that eosinophilic inflammation in bronchiectasis is not primarily Th2-driven and that another pathway through ILC2 cells possibly plays a role in eosinophilic inflammation; this has yet to be demonstrated, however. Furthermore, Tsikrika et al. do not provide data about circumstances such as the presence of atopy, which could impinge on the inflammatory phenotype and explain the eosinophilia found in these patients. The authors also point out that one of the limitations of their study is their inability to rule out the possibility that some of their subjects could have concomitant asthma.

In fact, few studies have assessed FeNo in subjects with asthma and bronchiectasis. In a recent retrospective study, Chen et al [61] measured FeNO levels in 99 patients with bronchiectasis (20 of them with asthma) and found higher FeNO levels in subjects with bronchiectasis and asthma, compared to subjects who had only bronchiectasis. The authors also demonstrated that FeNO levels can help distinguish patients with bronchiectasis and asthma from those with bronchiectasis but not asthma, and they established a cut-off point of 22.5 ppb, with an estimated AUC-ROC of 0.832. This is consistent with our results, as the optimal cut-off for FeNO levels that distinguished asthmatic subjects with bronchiectasis from asthmatic patients without bronchiectasis was 20.5 ppb, with a lower AUC-ROC. This is supported by the literature, since FeNO has been proposed by several guidelines [20, 62] for the diagnosis of asthma. In contrast, FeNO has not been proposed for the diagnosis of bronchiectasis, as FeNO levels in bronchiectasis are generally low [57], probably due to the prevalence of neutrophilic inflammation in these patients. According to the results obtained, FeNO’s most useful characteristic for the prognosis of bronchiectasis in asthma patients is its negative predictive value (81%).We found that FeNO levels can help rule out the presence of bronchiectasis since—as shown in Fig. 3a— asthmatic patients with high FeNO levels are unlikely to have bronchiectasis. Since FeNO is not effective in predicting the presence of bronchiectasis in asthmatic subjects (although it is effective for predicting its absence), we developed an innovative score with a good negative predictive value and excellent specificity. Thus, 95% of patients without bronchiectasis had a NOPES score of ≤2, and 76% of subjects with low scores did not have bronchiectasis, while 67% of patients with a high NOPES score had bronchiectasis. Furthermore, the likelihood of bronchiectasis rises as the NOPES score increases (according to the presence or absence of the four variables proposed). Low scores indicate the absence of bronchiectasis, whereas high scores suggest its presence.

As FeNO is not available in all centers, we made a score with three variables (excluding FeNO but retaining the other three variables), but it proved to be of little value in comparison with the score with four variables (AUC-ROC 0.648 and Nagelkerke’s R Square 0.08 vs AUC-ROC 0.7 and Nagelkerke’s R Square 0.145) and was therefore discarded.

We therefore propose the NOPES score as a clinically valuable, easy-to-use tool for predicting bronchiectasis in patients with uncontrolled asthma.

The strength of this study is its use of the largest prospective study of patients subjected to HRCT. It provides real data on the prevalence of bronchiectasis in patients with uncontrolled asthma, without biases such as smoking, ABPA, or other diseases causing bronchiectasis, as these conditions were excluded.

One limitation of this study, despite its large sample, is that patients were grouped according to the presence or absence of different variables, leaving some study arms with very few patients, as in the cases of subjects likely to develop bronchiectasis according to a NOPES score of 0% and 100%, where the highest *n* was 4; consequently, this probability can be underestimated in the former case and overestimated in the latter. Furthermore, induced sputum is unfortunately unavailable in our center, and so we were unable to establish our patients’ inflammatory phenotypes. Another limitation is that all the patients were treated in the same center, so a multicenter study would be required to confirm the results.

## Conclusions

The prevalence of bronchiectasis in UMSA is high. The severity of asthma, chronic expectoration, and a history of previous pneumonia can be independent predictive factors for bronchiectasis in subjects with UMSA, and high FeNO levels are related to a lower presence of bronchiectasis. We propose an easy-to-use predictive tool for bronchiectasis in these patients.

## Abbreviations

ABPA: Allergic bronchopulmonary aspergillosis
AUC: Area under the curve
FeNO: Fractional exhaled nitric oxide
FEV_1_: Forced expiratory volume in one second
FVC: Forced vital capacity
HRCT: High resolution computed tomography
NOPES: **N**itric oxide, **P**neumonia, **E**xpectoration, **S**everity
PA: *Pseudomonas aeruginosa*
ppb: Parts per billion
PPM: Potentially pathogenic microorganisms
ROC: Receiver operating characteristic
SD: Standard deviations
UMSA: Uncontrolled moderate-to-severe asthma
